# Swiprosin-1 modulates actin dynamics by regulating the F-actin accessibility to cofilin

**DOI:** 10.1007/s00018-013-1447-5

**Published:** 2013-08-21

**Authors:** Yun Hyun Huh, So Hee Kim, Kyoung-Hwun Chung, Sena Oh, Min-Sung Kwon, Hyun-Woo Choi, Sangmyung Rhee, Je-Hwang Ryu, Zee Yong Park, Chang-Duk Jun, Woo Keun Song

**Affiliations:** 1 Bio Imaging and Cell Dynamics Research Center, School of Life Sciences, Gwangju Institute of Science and Technology, Gwangju, 500712 Korea; 2 School of Biological Sciences, Joong Ang University, Seoul, 156756 Korea; 3 Research Center for Biomineralization Disorders and Dental Science Research Institute, School of Dentistry, Chonnam National University, Gwangju, 500757 Korea

**Keywords:** Swiprosin-1, Actin filament, Cofilin, Lamellipodia

## Abstract

Membrane protrusions, like lamellipodia, and cell movement are dependent on actin dynamics, which are regulated by a variety of actin-binding proteins acting cooperatively to reorganize actin filaments. Here, we provide evidence that Swiprosin-1, a newly identified actin-binding protein, modulates lamellipodial dynamics by regulating the accessibility of F-actin to cofilin. Overexpression of Swiprosin-1 increased lamellipodia formation in B16F10 melanoma cells, whereas knockdown of Swiprosin-1 inhibited EGF-induced lamellipodia formation, and led to a loss of actin stress fibers at the leading edges of cells but not in the cell cortex. Swiprosin-1 strongly facilitated the formation of entangled or clustered F-actin, which remodeled the structural organization of actin filaments making them inaccessible to cofilin. EGF-induced phosphorylation of Swiprosin-1 at Ser183, a phosphorylation site newly identified using mass spectrometry, effectively inhibited clustering of actin filaments and permitted cofilin access to F-actin, resulting in actin depolymerization. Cells overexpressing a Swiprosin-1 phosphorylation-mimicking mutant or a phosphorylation-deficient mutant exhibited irregular membrane dynamics during the protrusion and retraction cycles of lamellipodia. Taken together, these findings suggest that dynamic exchange of Swiprosin-1 phosphorylation and dephosphorylation is a novel mechanism that regulates actin dynamics by modulating the pattern of cofilin activity at the leading edges of cells.

**Electronic supplementary material:**

The online version of this article (doi:10.1007/s00018-013-1447-5) contains supplementary material, which is available to authorized users.

## Introduction

The membrane protrusions and lamellipodia necessary for cell migration have a highly dynamic actin structure that is characterized by the rapid turnover of actin filaments. These actin dynamics are regulated by a variety of actin-binding proteins that act cooperatively to mediate the assembly/disassembly and reorganization of actin filaments [1, 2]. Key regulators of actin dynamics include the Arp2/3 complex that initiates branching or polymerization of F-actin and ADF/cofilin that mediates severing or depolymerization of F-actin [3, 4] and modulates turnover of the actin network. In particular, cofilin binding changes the conformation of F-actin, weakening the lateral contacts between actin monomers [5–7], thereby initiating actin filament severance or depolymerization [8, 9]. Consequently, overactivation of cofilin would likely suppress lamellipodia extension by disrupting the actin filaments [10, 11]. But cofilin also contributes to the assembly of actin filaments by replenishing actin monomers for polymerization [1, 12, 13]. By severing actin filaments cofilin increases the availability of free barbed ends, which are the preferred substrates for nucleation by the Arp2/3 complex [3]. Appropriate fine-tuning of cofilin activity is thus crucial for proper membrane protrusion and lamellipodia formation.

The activity of cofilin appears to be mainly regulated by phosphorylation and dephosphorylation processes. Phosphorylation of cofilin at serine residue 3 (Ser3) is mediated by the LIM kinases (LIMK; Lin-11/Isl-1-1/Mec-3 kinases) [5, 14] and testicular protein kinases (TESK) [15, 16], which are regulated by Rho GTPases [17]. Upon phosphorylation, cofilin is detached from F-actin, and, consequently, its severing activity is abolished [18]. Dephosphorylation of Ser3 by slingshot [19] or chronophin [20] phosphatases reactivates cofilin. Additionally, Ca^2+^, cAMP and phosphoinositide 3-kinase have been shown to participate in the signaling pathway for cofilin dephosphorylation [21, 22]. For example, cellular Ca^2+^ increase by growth factors leads to activation of CaMKII and calcineurin, in turn, triggering Ca^2+^-dependent LIMK and/or SSH1L responsible for cofilin activation and subsequent actin cytoskeletal reorganization [23, 24]. However, actin-binding proteins also act in other ways to stabilize actin filaments against depolymerization by cofilin. For example, arabidopsis villin-1 prevents ADF/cofilin-mediated depolymerization by stabilizing actin filament bundles [25], while cross-linking proteins such as filamin and fascin inhibit cofilin-induced depolymerization of actin filaments by sterically hampering its access to the filaments [26]. On the other hand, actin filaments cross-linked by dynamin-2 and cortactin are dynamically unraveled in the presence of GTP, and the loosely remodeled filaments exhibit increased susceptibility to severing by cofilin [27]. There is still much that is not understood about how actin-binding proteins regulate the stability of actin filaments.

Swiprosin-1 (also known as EF hand domain containing 2; EFHD2) was first identified in human CD8+ lymphocytes [28]. The predicted domain structure of swiprosin-1 includes disordered regions at the N-terminus, followed by potential SH3-domain binding sites [29], two EF-hand domains and a coiled-coil domain at the C-terminus [30]. The presence of the two EF-hand domains in Swiprosin-1 suggests this protein could be regulated by Ca^2+^ signaling, and indeed Swiprosin-1 exhibits Ca^2+^-binding activity [31]. Because Swiprosin-1 was first identified in immune cells, its investigation has focused mainly on its involvement in immune responses [32, 33]. Recently, Ramesh et al. [34] demonstrated that Swiprosin-1 colocalizes with F-actin in HMC-1 human mast cells, and re-localizes into membrane apical ridges along with F-actin movement in PMA-treated Cos-7 cells, supporting the association of these two proteins. However, the functions of Swiprosin-1 in F-actin organization are still poorly understood.

In the present study, we demonstrate that Swiprosin-1 is involved in regulating the accessibility of F-actin to cofilin through the clustering of F-actin, and that the activity of Swiprosin-1 is highly dependent on its phosphorylation status at Ser183. The regulation of cofilin activity by Swiprosin-1 modulates membrane dynamics such as lamellipodia formation.

## Materials and methods

### Cell culture and plasmids

HEK293T and mouse melanoma B16F10 cells were grown in Dulbecco’s modified Eagle’s medium (Gibco-BRL, Grand Island, NY, USA) supplemented with 10 % (v/v) fetal bovine serum, 50 μg/ml streptomycin, and 50 units/ml penicillin. *hSwprosin*-*1* was constructed into the pLEGFP, pGEX4T1, and pCS2-myc vectors. Point mutations of S183 of Swiprosin-1 were accomplished by polymerase-chain reaction (PCR) site-directed mutagenesis. S183 was replaced with alanine or glutamic acid using the oligonucleotide primers 5′-gatcgacgtcgccagtgagggtg-3′ (for S183A) or 5′-gatcgacgtcgagagtgagggtg-3′ (for S183E) and its complement on the opposite strand. For the generation of short hairpin RNA against *Swiprosin*-*1*, the following two siRNA sequences were selected using siRNA Wizard (InvivoGen, San Diego, CA, USA): 5′-gagaagatgttcaagcagtat-3′ (sh1-1 and sh1-2, for both human and mouse *Swiprosin*-*1*) and, 5′-ctgcagtccacctttaagta-3′ (sh2-3 and sh2-4, for human-specific sequences). Complementary oligonucleotides for siRNAs, including restriction enzyme sites, 21-mer nucleotide sense, 7-mer hairpin loops (tcaagag), and 21-mer nucleotide antisense were designed according to the manufacturer’s specifications (InvivoGen). Annealed siRNAs were ligated into Acc65I/HindIII-digested psiRNA-hH1GFPzeo vector (InvivoGen). Cells were then transfected with the shRNAs using Lipofectamine 2000 (Invitrogen, Grand island, NY, USA).

### Antibodies

**Fig. 1:**
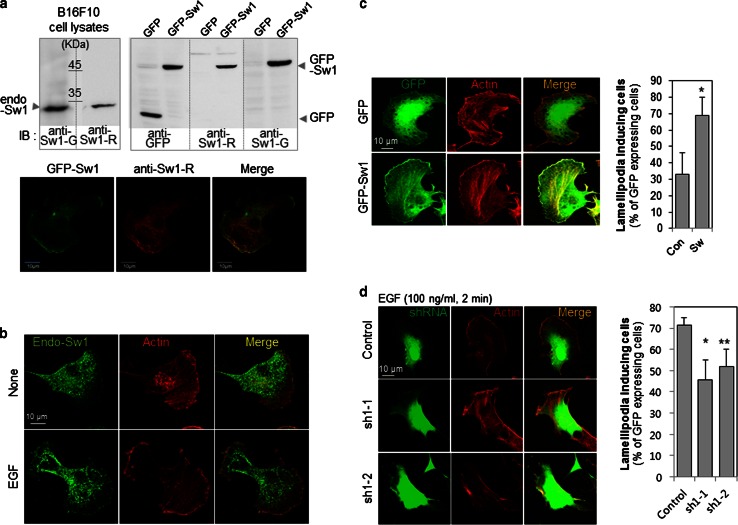
Swiprosin-1 colocalizes with F-actin and modulates lamellipodia formation. **a** Anti-Swiprosin-1 antibody (*Sw1-R Ab*) is a rabbit polyclonal antibody raised against recombinant human Swiprosin-1 protein. The antibody was compared with goat anti-Swiprosin-1 Ab (*anti-Sw1-G*) in IB experiments with B16F10 cells (*upper left panel*). Lysates were obtained from B16F10 cells transfected with empty *GFP* vector or *GFP*-*Swiprosin*-*1* for 24 h. GFP-Swiprosin-1 and transfection efficiency were detected with anti-Sw1-R, anti-Sw1-G, and anti-GFP antibodies (*upper right panel*). GFP signals merged with the anti-Sw1-R antibody staining (*lower panel*). **b** B16F10 cells were treated with or without 100 ng/ml EGF for 2 min, and stained with anti-Sw1-R antibody and phalloidin-594 to visualize F-actin. **c** B16F10 cells were transfected with empty *GFP* vector or *GFP*-*Swiprosin*-*1* for 24 h. Next, cells were stained with Alexa-594-phalloidin. *Merged images* are shown in *yellow*. *Scale bar* 10 μm. Lamellipodia formation was determined with actin staining among GFP-expressing cells. (*n* > 30 cells, *P < 0.005). **d** B16F10 cells were transfected with psi-RNA-*GFP*-control or psi-RNA-*GFP*-*Swiprosin*-*1* for 24 h, and 100 ng/ml EGF administered for 2 min for lamellipodia induction. The transfected cells were stained with Alexa-594-phalloidin, and the lamellipodia formation was analyzed in GFP-expressing cells (*P < 0.05, **P < 0.01)

Goat antibody against Swiprosin-1 (anti-Sw1-G), purchased from Imgenex (San Diego, CA, USA), was used for immunoblotting (IB). Anti-Sw1-R Ab is a rabbit polyclonal antibody raised against recombinant human Swiprosin-1 protein (full-length). Specificity of this antibody was tested using IB, immunoprecipitation (IP) (Supplementary Information, Fig. S1) and immunofluorescence (IF) staining (Fig. 1a). Anti-phospho-Ser (anti-pS) antibody was purchased from Millipore (Billerica, MA, USA). Anti-GFP (Santa Cruz Biotechnology, Santa Cruz, CA, USA), anti-cofilin (active) (Abcam, Cambridge, MA, USA), and anti-phospho-Ser3-cofilin (inactive) (Cell Signaling, Danvers, MA, USA) antibodies were utilized for IB.

### IP assay and IB analysis

Cells were lysed in modified radioimmunoprecipitation assay buffer (50 mM Tris–HCl, pH 7.4, 150 mM NaCl, 1 % NP-40, 0.25 % sodium deoxycholate, 10 mM NaF, 1 mM PMSF, 1 mM Na_3_VO_4_, 10 μM leupeptin, 1.5 μM pepstatin, and 10 μg/ml aprotinin), after which the lysates were cleared by centrifugation at 12,000 rpm for 10 min at 4 °C. The supernatant was then collected and, after addition of the appropriate antibody, it was incubated for 12 h at 4 °C, allowing the IP reaction to run. The resultant immune complexes were then collected by precipitation for 1 h with protein A or G-Sepharose (GE Healthcare, Piscataway, NJ, USA). After washing the mixtures three times with lysis buffer, the beads were suspended in SDS sample buffer, boiled for 5 min, and resolved by SDS-PAGE. For examining phosphorylation of endogenous Swiprosin-1 in B16F10 cells, anti-Sw1-R antibody was applied for IP, and IB performed with anti-pS or anti-Sw1-G antibody. Phosphorylation of Swiprosin-1 at Ser183 was analyzed based on its IP from pLEGFP-*Swiprosin*-*1 *(*WT*,* S183A* or* S183E*)-transfected HEK293T cells using anti-pS antibody, followed by IB with anti-GFP antibody. For analysis of the interaction of myc-Swiprosin-1 with cofilin, the lysates were immunoprecipitated with anti-myc antibody followed by IB with anti-cofilin (active) or anti-phospho-Ser3-cofilin (inactive) antibodies.

### IF staining and confocal imaging analysis

The cells cultured on fibronectin-coated glass covers were fixed with 4 % paraformaldehyde in PBS for 10 min at room temperature. The cells were then permeabilized with 0.1 % Triton X-100 for 10 min and blocked for 30 min with 1 % bovine serum albumin in PBS. Cells were incubated for 1 h with anti-Sw1-R antibody, followed by incubation for 1 h with Alexa 488-conjugated anti-rabbit secondary antibody (Molecular Probes, Eugene, OR, USA). The actin cytoskeleton was visualized using either Alexa 488- or Alexa 555-conjugated phalloidin (Molecular Probes). The glass coverslips were mounted using PermaFluor Aqueous Mountant (Thermo Scientific, Pittsburgh, PA, USA). Fluorescence images were obtained using an Olympus confocal microscope (FV1000) with FV10-MSASW software or with an Olympus IX81 Inverted fluorescence microscope driven by MetaMorph imaging software.

### In vitro actin co-sedimentation assay and actin bundling assay

To assemble F-actin, 5 μM monomeric G-actin (Cytoskeleton, Denver, CO, USA) purified from rabbit skeletal muscle was incubated in actin polymerization buffer containing 20 mM MgCl_2_, 0.5 M KCl, and 5 mM ATP for 30 min at 25 °C. Purified cofilin and/or GST-Swiprosin-1 fusion protein were added to the reaction at the indicated concentrations to verify the ability of Swiprosin-1 to inhibit the actin binding of cofilin. After incubation, the reaction mixtures were ultracentrifuged at 45,000 rpm for 1 h at 25 °C. The supernatants were transferred to new tubes, and the pellets were dissolved in SDS-loading buffer and subjected to SDS-PAGE, followed by staining with Coomassie brilliant blue R-250. For F-actin bundling assays, the protocol was the same except the ultracentrifugation step was replaced with centrifugation at 15,000 rpm for 10 min at 25 °C.

### In vitro actin polymerization and depolymerization assays

Actin polymerization and depolymerization activities were measured by monitoring the changes in fluorescence intensity of pyrene-labeled actin, as described previously [35]. Pyrene-labeled monomeric G-actin (Cytoskeleton) was dissolved in G-actin buffer (5 mM Tris–HCl, pH 8.0, 0.2 mM CaCl_2_, 0.5 mM dithiothreitol, and 0.2 mM ATP) and then left on ice for 1 h to allow depolymerization of any actin oligomers that formed during storage. The mixture was then centrifuged at 14,000 rpm for 30 min at 4 °C. GST-Swiprosin-1 or GST was then mixed with the pyrene-G-actin, and polymerization was initiated by adding the actin polymerization buffer. For actin depolymerization assays, pyrene-F-actin was pre-assembled, after which 5 μM F-actin was mixed with GST-Swiprosin-1-WT, the S183A or S183E mutants, or GST. Fluorescence levels were then recorded every 1 min for 35 min using a SpectraMax GEMINI XS spectrofluorometer (Molecular Devices, Sunnyvale, CA, USA). The excitation and emission wavelengths were 350 and 407 nm, respectively.

### Mass spectrometry

HEK293T cells were transfected with GFP-Swiprosin-1 and then treated with 100 ng/ml EGF for 5 min, after which proteins were immunoprecipitated using anti-GFP antibody on beads. For in-solution digestion, the purified proteins were eluted from the beads using two bed volumes of 9 M urea over the course of 1 h. The eluted protein samples were reduced by adding DTT to a final concentration of 5 mM, after which 25 mM iodoacetamide was added, and solutions were incubated for 30 min at room temperature. Then, after diluting the sample fourfold with 25 mM Tris–HCl (pH 8.2), sequencing grade trypsin (Promega, Madison, WI, USA) was added to an enzyme/substrate ratio of 1:50 (w/w), and the mixture was incubated overnight at 37 °C with rotation (400 rpm). Digestion was stopped by the addition of FA to 1 %. For enrichment of the phosphopeptides, the peptides were desalted and then applied to IMAC beads (PHOS-Select™ iron affinity gel; Sigma-Aldrich, St. Louis, MO, USA). The eluted peptides were loaded onto a trap column packed with 2 cm of AQUA C18 in a fused-silica capillary. Finally, a pre-conditioned analysis column (fused-silica capillary 100 μm i.d. × 360 μm o.d.; packed with 7 cm of AQUA C18) was connected to the trap column for micro-RPLC-MS/MS analysis. The analysis of the peptide samples was performed using an Agilent 1100 Series high-performance liquid chromatography (HPLC) pump (Agilent Technologies, Santa Clara, CA, USA) coupled to a linear quadruple ion trap mass spectrometer (LTQ; Thermo Finnigan, San Jose, CA, USA) using a nanoESI interface manufactured in-house. The 10 most abundant ions from each MS scan were selected for further MS/MS analysis using normalized collision energy of 35 %. Dynamic exclusion for 30 s was applied to avoid repeated analysis of the same abundant precursor ion. The SEQUEST algorithm was used to search a composite database containing the mouse IPI protein database (v.3.28) and its reversed complement.

### Transmission electron microscopy

Skeletal muscle actin (5 μM) was polymerized in actin polymerization buffer containing GST, GST-Swiprosin-1, or a Swiprosin-1 mutant (S183A or S183E). After incubation for 1 h at 25 °C, the reaction mixtures were adsorbed onto formvar/carbon-coated copper grids for 30 s, and the excess sample was removed by wicking with filter paper. The bound actin was stained with 1 % uranyl acetate for 1 min, and the excess stain was removed with filter paper. Images were recorded on a FEI TECNAI G^2^ transmission electron microscope operated at 120 kV.

## Results

### Swiprosin-1 colocalizes with F-actin and modulates lamellipodia formation

We initially generated rabbit anti-Swiprosin-1 antibody (anti-Sw1-R) against recombinant human Swiprosin-1 protein (full-length) and examined antibody specificity (Supplementary Fig. 1). Overexpression of GFP-Swiprosin-1 in mouse melanoma B16F10 cells was detected via immunoblot analysis. The GFP signal of GFP-Swiprosin-1 overlapped exactly with that of the anti-Sw1-R antibody stain, confirming the membrane localization of Swiprosin-1 (Fig. 1a). To examine the roles of Swiprosin-1 in membrane dynamics, B16F10 cells were treated with EGF. Upon EGF treatment, lamellipodia formation was markedly induced and endogenous Swiprosin-1 was rapidly translocated to the lamellipodia which exhibited local enrichment of phalloidin-stained F-actin (Fig. 1b). In addition, lamellipodia formation was well detected in Swiprosin-1 overexpressed B16F10 cells (Fig. 1c). To further confirm the function of Swiprosin-1 in lamellipodia formation, the protein was knocked down using shRNAs targeting specific Swiprosin-1 regions conserved in human and mouse (sh1-1 and sh1-2 clones) or specifically in human (sh2-3 and sh2-4) (see Supplementary information, Fig. S2). The knockdown of Swiprosin-1 was verified by RT-PCR, qRT-PCR and western blotting, and it had no effect on the expression of Swiprosin-2, an isoform of Swirpsoin-1 with over 70 % amino acid sequence homology (see Supplementary information, Fig. S2). Within the Swiprosin-1 knockdown cells, based on their GFP expression, actin stress fibers were prominent in the cell cortex but not at the leading edge, and EGF-induced lamellipodia formation was significantly inhibited (Fig. 1d). To further clarify the role of Swiprosin-1 in lamellipodia formation, we used kymograph analysis to assess lamellipodia dynamics in cells overexpressing GFP-Swiprosin-1. Our findings showed that the dynamic GFP-Swiprosin-1 signal moved concurrently with the leading edge of the lamellipodia, by contrast, the movement of GFP (negative control) was unrelated to the lamellipodia dynamics (see Supplementary information, Fig. S3). Collectively, these data indicate that Swiprosin-1 modulates lamellipodia formation and actin structure at the leading edges of cells.

### Swiprosin-1, a novel actin-binding protein, inhibits cofilin activity by preventing the F-actin accessibility to cofilin

**Fig. 2:**
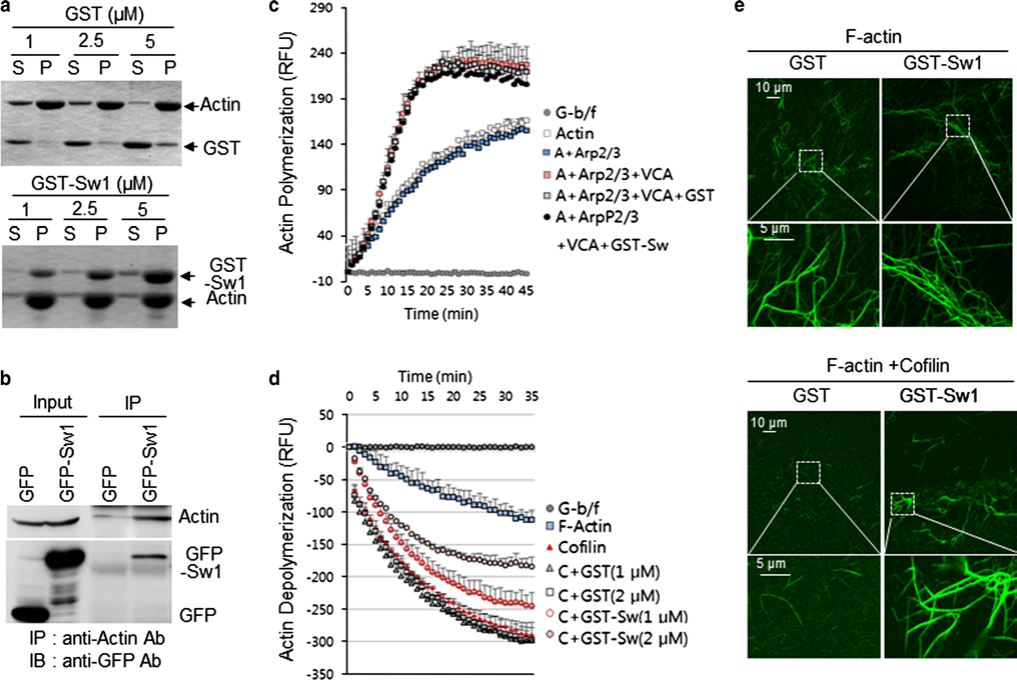
Swiprosin-1 inhibits cofilin-mediated actin depolymerization. **a** The indicated concentrations of purified GST or GST-Swiprosin-1 were incubated with monomeric G-actin (5 μM) for 30 min to assemble actin filaments and then centrifuged at 45,000 rpm for 2 h. The supernatant (*S*) and pellet (*P*) fractions were then separated by SDS-PAGE. **b** B16F10 cells were transfected with empty *GFP* vector or *GFP*-*Swiprosin*-*1* for 24 h. Cell lysates were applied for immune-precipitation with anti-actin antibody and for IB with anti-actin and anti-GFP antibodies. Effects of Swiprosin-1 on actin polymerization or depolymerization were assessed by pyrene-actin assays. The kinetics was monitored by measuring the fluorescence intensity of pyrene-F-actin. **c** The reaction mixture for actin polymerization assay contained 5 μM pyrene-actin, 50 nM Arp2/3 complex, 50 nM WASP-VCA, and 1 μM GST or GST-Swprosin-1. **d** For cofilin-mediated actin depolymerization, pre-polymerized pyrene-F-actin (5 μM) in the presence of cofilin (250 nM) and the indicated concentrations of GST or GST-Swiprosin-1. Actin alone served as a control. **e** G-actin (5 μM) was incubated with 1 μM GST or GST-Swiprosin-1 for 30 min at room temperature to assemble F-actin. A half volume of reaction was subsequently stained with Alexa-488-phalloidin. The other half was incubated with 250 nM cofilin for 1 h to disassemble F-actin and then stained with Alexa-488-phalloidin. The reactions were observed under a confocal microscope

The findings that Swiprosin-1 colocalizes with F-actin and regulates lamellipodia formation at the cell leading edge led us to examine the effects of Swiprosin-1 on actin polymerization. We determined whether Swiprosin-1 binds to F-actin directly using in vitro actin co-sedimentation assay. Our data showed that GST-Swiprosin-1 binds directly to F-actin in vitro, but not GST (Fig. 2a). The finding that actin was coimmunoprecipitated with Swiprosin-1 in GFP-Swiprosin-1 overexpressed cells further supports the interaction between Swiprosin-1 and actin (Fig. 2b). When we assayed the polymerization of pyrene-labeled actin in the presence of GST-Swiprosin-1 in vitro, we found that Swiprosin-1 did not promote actin polymerization mediated by the Arp2/3 complex and VCA domain of WASP, a cofactor of Arp2/3 complex (Fig. 2c), and did not function as a cofactor like VCA domain (see Supplementary information, Fig. S4). Instead, Swiprosin-1 was remarkably effective at inhibiting cofilin-mediated actin depolymerization (Fig. 2d). The inhibition was dose-dependent, and 2 μM GST-Swiprosin-1 elicited ~80 % inhibition of cofilin-mediated actin depolymerization. To further confirm these observations, the actin used in the depolymerization assay was stained with phalloidin-488 and observed under a confocal microscope (Fig. 2e). In the presence of Swiprosin-1, the F-actin was found in clustered or entangled filaments that were not seen in the absence of Swiprosin-1 (upper panel). Moreover, the entangled actin filaments were retained even in the presence of cofilin when Swiprosin-1 was also present (lower panel), whereas extensive cofilin-mediated actin depolymerization occurred in the absence of Swiprosin-1, which eliminated nearly all of the F-actin (Fig. 2e). This suggests Swiprosin-1 inhibits cofilin-mediated actin depolymerization.

**Fig. 3:**
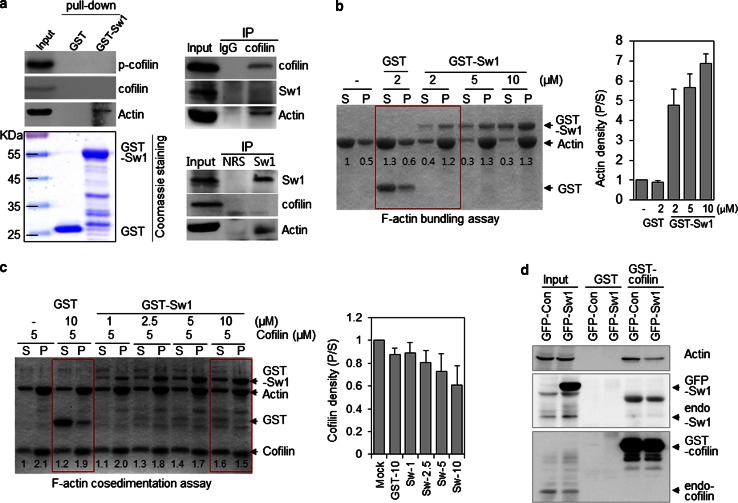
Swiprosin-1 inhibits cofilin activity by preventing its access to F-actin. **a** In vitro binding assay carried out with GST or GST-Swiprosin-1 agarose beads using B16F10 cell lysates (*left panel*). Lysates of B16F10 cells were immunoprecipitated with anti-cofilin (*upper*) and anti-Sw1-R (*lower*) antibodies (*right panel*). The bound proteins were verified using anti-cofilin, anti-phospho-cofilin (Ser3), anti-Sw1-R, and anti-actin antibodies. The presence of GST and GST-Swiprosin-1 was confirmed by coomassie blue staining. **b** The indicated concentrations of GST or GST-Swprosin-1 were incubated with 5 μM G-actin for 1 h and then subjected to F-actin bundling assay. **c** Competition between Swiprosin-1 and cofilin for F-actin binding was assessed in F-actin co-sedimentation assay. Preassembled F-actin (5 μM) and cofilin were incubated with 10 μM GST or the indicated concentrations of GST-Swiprosin-1. The reactants were co-sedimented and the supernatant (*S*) and pellet (*P*) fractions were separated by SDS-PAGE. Cofilin band densities were measured by densitometry and the pellet-to-supernatant ratio is shown. **d** Lysates of B16F10 cells transfected with *GFP*-*Ev* or *GFP*-*Swiprosin*-*1* were pulled down using GST or GST-cofilin agarose beads and immunoblotted with anti-actin, anti-Sw1-R, or anti-cofilin antibodies

To investigate the mechanism underlying the inhibitory effect of Swiprosin-1 on cofilin activity, we carried out in vitro GST-Swiprosin-1 pull-down assay (Fig. 3a) and IP assay using lysates from cells overexpressing myc-tagged Swiprosin-1 (see Supplementary information, Fig. S5). No interactions between endogenous cofilin and Swiprosin-1 were observed in B16F10 cells with the IP assay (Fig. 3a, right panel). We found that Swiprosin-1 does not bind to either inactive (phosphorylated at Ser3) or active (unphosphorylated) cofilin. Recently, it was reported that actin filament cross-linking or bundle formation mediated by actin-binding proteins such as fascin and tropomyosin protects actin filaments from depolymerization by cofilin [26]. Based on these findings, we performed actin bundling sedimentation assay to examine whether Swiprosin-1 induces F-actin clustering. Actin bundling assay showed that Swiprosin-1 acted dose-dependently to induce F-actin clustering (Fig 3b). We therefore hypothesized that Swiprosin-1-mediated F-actin clustering denies cofilin access to F-actin. To test this hypothesis, we initially performed F-actin co-sedimentation assay using a single concentration of cofilin and several concentrations of GST-Swiprosin-1. Our experiment revealed that, with increasing concentrations of Swiprosin-1, the amount of cofilin bound to F-actin in the pellet decreased (Fig. 3c). Conversely, when the assay was carried out using a single concentration of Swiprosin-1 and serially increasing concentrations of cofilin, Swiprosin-1 binding to F-actin gradually declined as actin filament depolymerization, caused by cofilin activity, was increased (see Supplementary information, Fig. S6). In addition, the amount of actin pulled down by GST-cofilin beads was dramatically reduced by overexpression of Swiprosin-1 (Fig. 3d), suggesting Swiprosin-1 inhibits cofilin activity by blocking its access to F-actin.

### EGF induces phosphorylation of Swiprosin-1 at Ser183

**Fig. 4:**
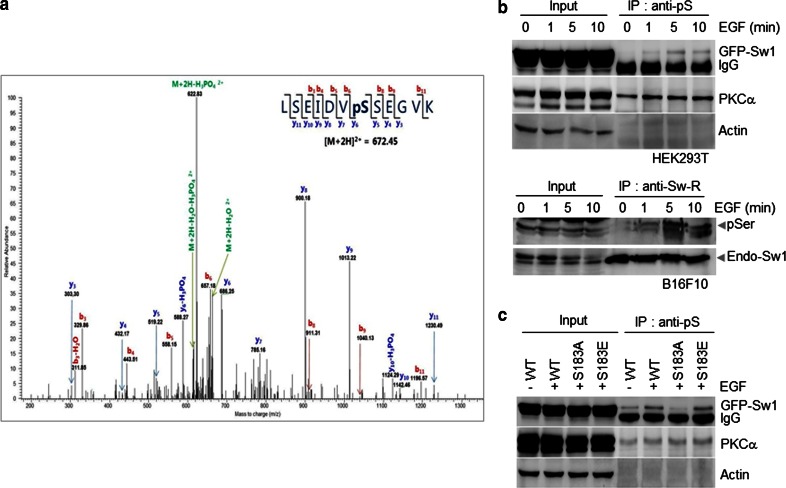
EGF induces phosphorylation of Swiprosin-1 at Ser183. **a** HEK293T cells transfected with *GFP*-*Swiprosin*-*1* were exposed to 100 ng/ml EGF for 5 min, after which the lysate was immunoprecipitated with anti-GFP antibody. Phosphorylation of Swiprosin-1 was analyzed by mass spectrometry**. b** HEK293T cells transfected with GFP-tagged *Swiprosin*-*1* and B16F10 cells were exposed to 100 ng/ml EGF for the indicated times. Lysates were subsequently immunoprecipitated with anti-pSer or anti-Sw1-R antibody, respectively. **c** HEK293T cells transfected with GFP-tagged *Swiprosin*-*1*-WT, S183A, or S183E mutants were exposed to EGF for 5 min. Lysates were immunoprecipitated using anti-pS and immunoblotted with anti-GFP, anti-PKCɑ antibodies (positive control for the EGF signaling), or anti-actin antibody (internal control)

Swiprosin-1 reportedly exhibits the same activation profile as Arp2/3 complex and Gelsolin in cells treated with EGF, suggesting a potential role in growth factor-dependent actin remodeling [36]. We therefore used mass spectrometry to investigate the mechanism of EGF-dependent activation of Swiprosin-1. To investigate the physiological functions of Swiprosin-1 and related human diseases based on regulation of actin dynamics, we employed HEK293T cells to screen for phosphorylation of Swiprosin-1 from the normal human database. HEK293T cells transfected with GFP-Swiprosin-1 were treated with EGF for 5 min, and then lysed and immunoprecipitated with anti-GFP antibody. The bound proteins were digested with trypsin, and the peptide fragments were analyzed by mass spectrometry, which revealed that Ser183 of Swiprosin-1 is a potential site for EGF-induced phosphorylation (Fig. 4a). EGF-mediated phosphorylation of Swiprosin-1 at serine was transient and reached peak levels at 5 min after EGF stimulation in both B16F10 and GFP-Swiprosin-1-transfected HEK293T cells (Fig. 4b). This finding was further confirmed with site-directed mutagenesis experiments whereby Ser183 was substituted with alanine (S183A, phosphorylation-deficient form) or glutamate (S183E, phosphorylation-mimicking mutant) (Fig. 4b, c).

### Inhibition of cofilin by Swiprosin-1 is dependent on the phosphorylation status of Swiprosin-1

**Fig. 5:**
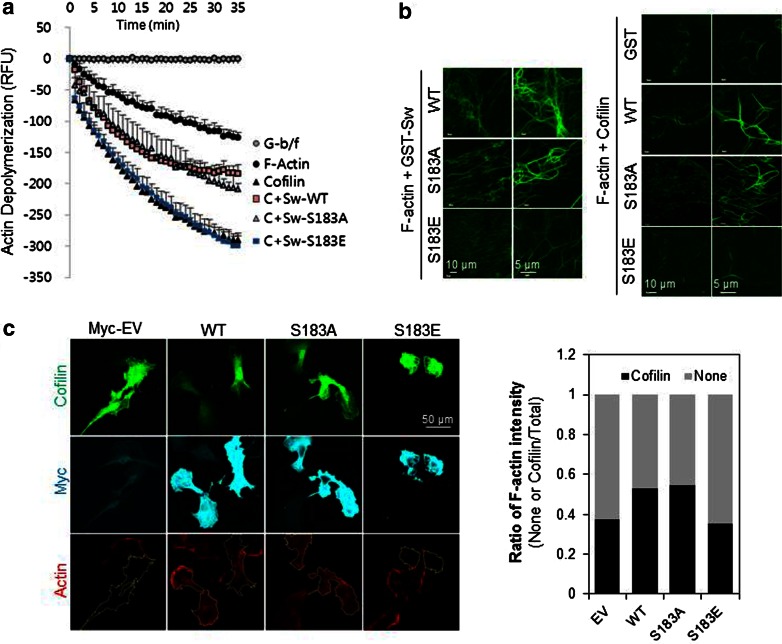
Inhibition of cofilin by Swiprosin-1 is dependent on the phosphorylation status of Swiprosin-1. **a** Actin depolymerization in a reaction mixture containing pre-polymerized pyrene-F-actin (5 μM) and 1 μM GST, GST-Swiprosin-1-WT, GST-Sw-S183A, or GST-Sw-S183E was initiated by addition of cofilin (250 nM). Traces show the time course of the actin depolymerization. **b** G-actin (5 μM) was incubated with 1 μM GST, GST-Sw-WT, GST-Sw-S183A, or GST-Sw-S183E for 30 min at room temperature to assemble F-actin, after which a half volume of reaction was stained with Alexa-488-phalloidin. The other half was incubated with 250 nM cofilin for 1 h to disassemble F-actin and then stained with Alexa-488-phalloidin. The reactions were observed under confocal microscope. **c** B16F10 cells were co-transfected with *GFP*-*cofilin* and myc-tagged *Swiprosin*-*1*-WT or the S183A or S183E mutant. Cofilin activity was visualized in individual cells after immunofluorescent staining with alexa-594-conjugated phalloidin

As shown in Fig. 2d, recombinant Swiprosin-1 inhibited cofilin-mediated actin depolymerization. To determine whether the inhibition was affected by the phosphorylation status of Swiprosin-1 at Ser183, we performed in vitro actin depolymerization assay using Swiprosin-1-WT and the S183A and S183E mutants. Swiprosin-1-WT and the phosphorylation-deficient S183A mutant each inhibited cofilin-induced actin depolymerization, whereas S183E, a phosphorylation-mimicking mutant, did not (Fig. 5a). These results were confirmed by microscopic examination of phalloidin-stained F-actin. Swiprosin-1-WT and the S183A mutant each induced formation of entangled or clustered actin filaments that persisted despite incubation with cofilin. By contrast, the S183E mutant induced a loose actin structure and nearly all the filaments were disassembled when incubated with cofilin (Fig. 5b).

**Fig. 6:**
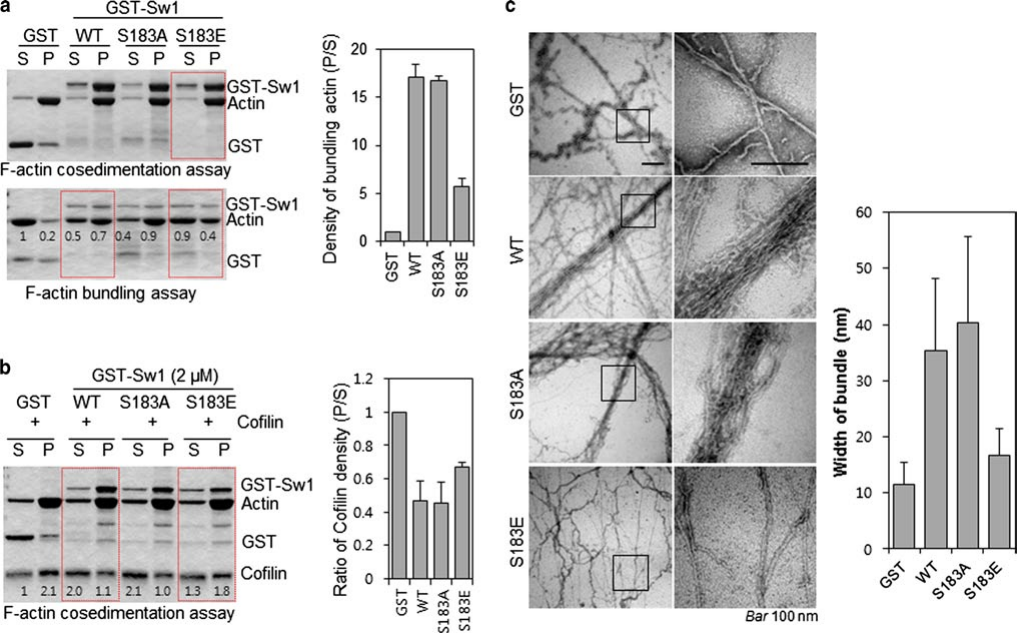
Phosphorylated Swiprosin-1 at S183 fails to promote F-actin clustering and permits access of cofilin to F-actin. Actin polymerization was induced using the indicated proteins. **a** The resultant F-actin was subjected to co-sedimentation (*upper panel*) or actin bundling (*lower panel*) assays. **b** The same assays were run after the polymerized actin was incubated with 2 μM cofilin for 30 min. **c** Reaction mixtures were adsorbed onto carbon-coated grids and stained with 1 % uranyl acetate for 1 min. Electron micrographs were obtained at magnifications of ×26,500 and ×135,000. *Bar* 100 nm. Width of clustered F-actin (*n* > 100) was quantified using MetaMorph software

**Fig. 7:**
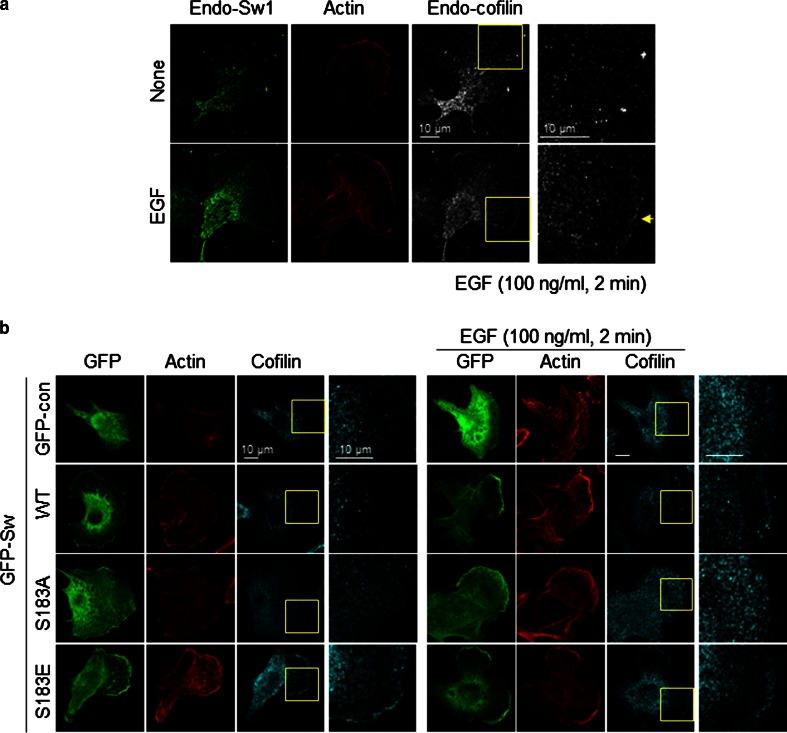
Phosphorylation of Swiprosin-1 at S183 modulates translocation of cofilin at the lamellipodial edge. **a** B16F10 cells grown on glass covers were stimulated with 100 ng/ml EGF for 2 min and stained with anti-Sw1-R and anti-cofilin antibodies following treatment with Alexa Fluor-488 and -647 dyes, respectively. Finally, F-actin was stained with Alexa-594-conjugated phalloidin and examined under a confocal microscope. **b** B16F10 cells transfected with GFP-Sw1 WT, S183A, S183E, or GFP-control vector were stimulated with EGF and stained with anti-cofilin antibody and Alexa-594-phalloidin

To further confirm the phosphorylation dependency of Swiprosin-1-mediated inhibition of cofilin activity, we next examined the F-actin content of cells. IF analysis revealed that, in cells overexpressing GFP-cofilin alone or together with S183E, phalloidin-stained F-actin was either absent or small amounts remained in a loose form. By contrast, F-actin was prominent in cells co-expressing GFP-cofilin with myc-tagged Swiprosin-1-WT or -S183A (Fig. 5c). Interestingly, phosphorylation of Swiprosin-1 at Ser183 significantly inhibited F-actin clustering without changing the binding affinity of Swiprosin-1 for F-actin (Fig. 6a). Moreover, cofilin binding to F-actin in the presence of the phosphorylation-mimetic S183E Swiprosin-1 mutant was enhanced, compared to that in the presence of wild-type Swiprosin-1 (WT) or the S183A mutant (Fig. 6b). The data suggest that Swiprosin-1 phosphorylation at Ser183 causes production of a loose form of F-actin by preventing F-actin clustering, thereby enhancing the accessibility of F-actin to cofilin. Transmission EM images confirmed that Swiprosin-1-WT or -S183A induced dense and thick actin filaments, whereas the S183E mutant induced loose and thin actin filaments (Fig. 6c). Next, we examined cofilin distribution at the leading edges of lamellipodia. Following EGF stimulation, endogenous cofilin was clearly detected at the leading edges of lamellipodia, with co-localization of Swiprosin-1 (Fig. 7a). Interestingly, cofilin translocation was prominent in cells expressing the Swiprosin-1 S183E mutant, but abolished in those with the S183A mutant, even after EGF stimulation (Fig. 7b). The data indicate that phosphorylation of Swiprosin-1 modulates cofilin distribution to the leading edges of lamellipodia induced by EGF. Based on the collective findings, we suggest that Swiprosin-1 modulates the accessibility of F-actin to cofilin in a manner highly dependent on phosphorylation of Swiprosin-1 at Ser183.

### Dynamic exchange of phosphorylation/dephosphorylation of Swirosin-1 is a key modulator of lamellipodia dynamics

**Fig. 8:**
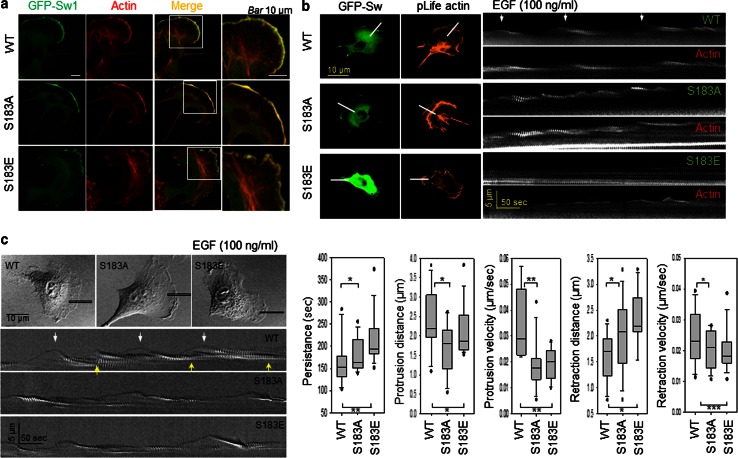
Dynamic exchange of phosphorylation/dephosphorylation of Swiprosin-1 is a key regulator of lamellipodia dynamics. **a** B16F10 cells transfected with *GFP*-*Swiprosin*-*WT*, -*S183A*, or -*S183E* constructs were stained with Alexa-594-phalloidin. *Scale bar* 10 μm. Kymographs were obtained from time-lapse images captured every 5 s for 10 min after addition of 100 ng/ml EGF. *Scale bar* 10 μm. **b** Dynamics of GFP-Swiprosin-WT, -S183A, or -S183E, and pLife-Actin are shown. The repeat pattern is indicated by *arrows*. **c** Lamellipodia dynamics were quantified using stroboscopic analysis of cell dynamics (SACED). Stroboscopic images were produced using a *five-pixel-wide box* drawn in the direction of the cell protrusion. The repeat pattern is indicated by *arrows*. *Box* and *whisker plots* for the persistence, protrusion distance, protrusion velocity, retraction distance, and retraction velocity of individual events (*n* > 10). The *middle line* in each *box* indicates the median, the *top* of each *box* indicates the 75th percentile, the *bottom* indicates the 25th percentile, and the *whiskers* indicate the extent of the 10th and 90th percentiles, respectively. (*P < 0.05, **P < 0.01, ***P < 0.005)

We next examined whether the phosphorylation status of Swiprosin-1 affects membrane dynamics such as lamellipodia formation. When overexpressed, GFP-Swiprosin-1 WT was enriched at lamellipodia, where it colocalized with actin. Likewise, S183A and S183E were also readily distributed to the cell leading edges (Fig. 8a). However, different shapes of membrane edges were observed. Smooth edges of membrane were observed in S183A-expressed cells while meandering edges were in S183E-expressed cells. Swiprosin-1 WT-expressed cells showed both shapes. The clear difference between the smooth and meandering edge shapes enabled us to analyze the membrane dynamics. Kymographic analysis then revealed that the cycles of protrusion and retraction of the WT-membrane were relatively regular and exhibited a regular pattern of repeats (Fig. 8b, c, arrows), during which levels of GFP-Swiprosin and F-actin were well correlated with the cycle of protrusion and retraction. By contrast, the movement of S183A- or S183E-membranes was extremely irregular (Fig. 8b, c), indicating that the membrane dynamics was less well coordinated than in cells expressing Swiprosin-1-WT. Lamellipodia dynamics were quantified using stroboscopic analysis of cell dynamics (SACED) (Fig. 8c). The average persistence time of Swiprosin-1-WT (151.40 ± 34.14 s) in overexpressing cells was shorter than that of S183A (168.10 ± 25.30 s) or S183E (195.08 ± 25.43 s). WT cells showed longer lamellipodial protrusion distances (2.38 ± 0.75 μm) and faster protrusion velocities (0.0297 ± 0.0124 μm/s) than S183A (distance: 1.82 ± 0.79 μm, velocity: 0.0187 ± 0.0094 μm/s) or S183E (distance: 1.94 ± 0.37 μm, velocity: 0.0258 ± 0.0113 μm/s) cells. The retraction distance of WT cells was shorter (1.68 ± 0.52 μm), and their velocity was faster (0.0242 ± 0.0089 μm/s) than S183A (distance: 2.09 ± 0.62 μm, velocity: 0.0199 ± 0.0062 μm/s) or S183E (distance: 2.33 ± 0.53 μm, velocity: 0.0202 ± 0.0068 μm/s) cells. The shorter protrusion distances and slower protrusion velocities of S183A- and S183E-expressing cells reflected the dysregulation of membrane dynamics following uncontrol of actin dynamics. In other words, the balance of actin dynamics is crucial for determining membrane dynamics such as the assembly and disassembly of lamellipodia. Taken together, these data support the idea that dynamic exchange of phosphorylation/dephosphorylation of Swiprosin-1 plays a key role in regulating membrane dynamics.

## Discussion

Lamellipodial protrusion is the first step in cell movement and is dependent on forces generated through actin dynamics at the plasma membrane [37, 38]. Cofilin is the best-known regulator of actin dynamics. It acts by severing and depolymerizing actin filaments, which increases the availability of free barbed F-actin ends [3] and G-actin monomers [39]. Appropriate regulation of cofilin activity is thus crucial for proper actin turnover.

Here, we demonstrated that overexpression or knockdown of Swiprosin-1 alters lamellipodia formation in B16F10 cells. The B16F10 cell line has been generally used for the lamellipodia dynamic assay, since it is a highly motile malignant cancer cell line. The mechanism underlying these effects appears to involve regulation of cofilin activity through modulation of F-actin clustering, which is dependent on the phosphorylation status of Swiprosin-1. Swiprosin-1 directly binds to F-actin and dose-dependently inhibits cofilin-mediated actin depolymerization by preventing the binding of cofilin to F-actin. Because there was no evidence of direct binding of Swiprosin-1 to cofilin, we suggest that, by mediating F-actin clustering, Swiprosin-1 reduces the accessibility of F-actin to cofilin.

It was previously shown that actin-binding proteins such as filamin and fascin sterically hinder the access of cofilin to actin filaments [26], and that enhancement of actin bundling by villin-1 prevents cofilin-mediated actin depolymerization [25]. On the other hand, when actin bundles induced by dynamin 2 and cortactin were remodeled to a more loosely packed conformation in the presence of GTP, the actin became more susceptible to depolymerization by cofilin [27]. Consistent with these earlier observations, we found that Swiprosin-1-mediated F-actin clustering inhibited cofilin binding to F-actin (Fig. 3d), so that F-actin staining was prominent in cells co-expressing cofilin and Swiprosin-1 (Fig. 5c). Apparently, F-actin clustering by Swiprosin-1 modulates susceptibility of actin filaments to depolymerization by cofilin.

We ruled out the possibility of competition between Swiprosin-1 and cofilin for F-actin binding with the aid of several experiments (Fig. 6a, b; Supplementary Fig. S6). Notably, only the S183E mutation facilitated cofilin access to F-actin by loosening the actin bundle, even though both phospho-mimetic (S183E) and phospho-deficient (S183A) mutants bound to F-actin. However, the potential involvement of other factors known to regulate cofilin activity, such as ATP, pH, calcium concentration, or other regulatory proteins (LIMK or slingshot) [21], cannot be overlooked. It is possible that single actin filaments alter their torsional twist upon Swiprosin-1 binding or that filaments cross-linked by Swiprosin-1 are less flexible than free filaments and therefore impacted on cofilin binding. Moreover, the theory that actin structure is changed by Swiprosin-1 remains to be examined.

The phosphorylation of Swiprosin-1 was predicted by an earlier report showing that Swiprosin-1 exhibits the same dynamics as Arp2/3 complex and Gelsolin in profiles of tyrosine phosphorylation [36]. For that reason, we initially focused on the tyrosine phosphorylation. Swiprosin-1 contains two tyrosine residues, Tyr83 and Tyr104, with the latter situated in the first of Swiprosin-1′s two EF-hand motifs. However, substitution of the residues confirmed that tyrosine phosphorylation is irrelevant to actin assembly/disassembly in vitro (data not shown). Instead, mass spectrometric analysis identified Ser183 as a novel site subject to EGF-induced phosphorylation. Phosphorylation of Swiprosin-1 at Ser183 did not affect its binding to actin filaments nor distribution of Swiprosin-1 at the lamellipodia, but Ser183 phosphorylation did block Swiprosin-1′s ability to inhibit cofilin activity. Kymographic analysis of the membrane dynamics showed that dynamic exchange of Swiprosin-1 phosphorylation and dephosphorylation is an essential process regulating the actin dynamics mediating lamellipodia formation. Similarly, phosphorylation of fascin at Ser39 by protein kinase C (PKC) inhibits F-actin bundling and alters actin reorganization [40, 41], though the regulatory function of this effect is not known. In our study, these phenomena appear to be associated with the regulation of cofilin activity at the membrane leading edge.

The relationship between cofilin-modulated F-actin dynamics and the regulation of the coordinated protrusion and retraction of lamellipodia is complex. Localized activation of cofilin has been shown to promote membrane protrusion at the local edge [42], and this behavior is explained by cofilin’s ability to generate barbed ends and replenish the pool of actin monomers. LIMK1- and SSH1L-mediated spatiotemporal regulation of cofilin activity is critical for polarized lamellipodia formation [43]. However, undue increases in cofilin activity could disrupt the balance between local protrusion and retraction along the edges of lamellipodia by altering F-actin kinetics [44]. We suggest that the phosphorylation/dephosphorylation of Swiprosin-1 at the leading edges of lamellipodia controls the F-actin clustering and thereby regulates the activity of cofilin by modulating the F-actin accessibility to cofilin. In our experiments, endogenous and overexpressed cofilin displayed translocation to the leading edges of lamellipodia after EGF stimulation, which was abolished in cells expressing the S183A mutant (Fig. 7a, b). Thus, phosphorylation of Swiprosin-1 may be one of the steps of spatial and temporal regulation of cofilin activity at the leading edges of lamellipodia of moving cells. Consistent with that idea, we found that only Swiprosin-1-WT, which is subject to both phosphorylation and dephosphorylation, supported regular patterns of membrane dynamics along with coordinated actin movement. Neither the S183E mutant nor the S183A mutant supported appropriate actin dynamics. The dysregulation of membrane dynamics may result in the different patterns of membrane edges such as smooth edges for the phosphorylation-deficient mutant (S183A) and meandering patterns for the phosphorylation-mimicking mutant (S183E).

Swiprosin-1 contains 2 EF-hand domains that bind to Ca^2**+**^, and cofilin activity is modulated by Ca^2+^ signaling. Ca^2+^-induced cofilin dephosphorylation is mediated by two protein phosphatases, calcineurin and SSH1L [23]. For example, soluble growth factors, such as EGF, activate the PLC-IP_3_-Ca^2+^-calcineurin signaling pathway, followed by transient increase of cofilin at the leading edge for neurite outgrowth and MTLn3 cancer cell migration [45–47]. EGF-simulated phosphorylation of Swiprosin-1 leads to loosening of F-actin bundles, thereby increasing the accessibility of F-actin to cofilin at the leading edge. Recruitment of cofilin to the leading edge results in the generation of short actin filaments with free barbed ends, which may, in turn, facilitate actin dynamics. At present, however, the molecular mechanism by which phosphorylation/dephosphorylation of Swiprosin-1 is regulated at the leading edges of cells remains uncertain, as does the mechanism by which conformational changes in F-actin are differentially induced through phosphorylation of Swiprosin-1.

Inhibition of PKC by GF109203X blocked EGF-induced phosphorylation of Swiprosin-1 at Ser183 (data not shown). We suggest that PKC may be an upstream kinase catalyzing Swiprosin-1 phosphorylation. At present, however, the molecular mechanism by which phosphorylation/dephosphorylation of Swiprosin-1 is regulated at the leading edges of cells remains uncertain, as does the mechanism by which conformational changes in F-actin are differentially induced through phosphorylation of Swiprosin-1.

In summary, we demonstrated here that the signaled (e.g., by EGF) dynamic exchange of Swiprosin-1 phosphorylation and dephosphorylation constitutes a novel mechanism that modulates the F-actin accessibility to cofilin by regulating the stability of F-actin at the leading edges of cells.

## Electronic supplementary material

Below is the link to the electronic supplementary material.

Supplementary material (DOCX 806 kb)

## References

[1] Pollard TD, Borisy GG (2003). Cellular motility driven by assembly and disassembly of actin filaments. Cell.

[2] Revenu C, Athman S, Robine S, Louvard D (2004). The co-workers of actin filaments: from cell structures to signals. Nat Rev Mol Cell Biol.

[3] Yamaguchi H, Chondeelis J (2007). Regulation of the actin cytoskeleton in cancer cell migration and invasion. Biochim Biophys Acta.

[4] Mouneimne G, DesMarais V, Sidani M, Scemes E, Wang W, Song X, Eddy R, Condeelis J (2006). Spatial and temporal control of cofilin activity is required for directional sensing during chemotaxis. Curr Biol.

[5] McGough A, Chiu W (1999). ADF/cofilin weakens lateral contacts in the actin filament. J Mol Biol.

[6] Bobkov AA, Muhlrad A, Shvetsov A, Benchaar S, Scoville D, Almo SC, Reisler E (2004). Cofilin (ADF) affects lateral contacts in F-actin. J Mol Biol.

[7] Paavilainen VO, Okasanen E, Goldman A, Lappalainen P (2008). Structure of the actin-depolymerizing factor homology domain in complex with actin. J Cell Biol.

[8] Prochniewicz E, Janson N, Thomas DD, De la Cruz EM (2005). Cofilin increases the torsional flexibility and dynamics of actin filaments. J Mol Biol.

[9] McCullough BR, Blanchoin L, Martiel JL, De la Cruz EM (2008). Cofilin increases the bending flexibility of actin filaments: implications for severing and cell mechanics. J Mol Biol.

[10] Iwasa JH, Mullins RD (2007). Spatial temporal relationships between actin-filament nucleation capping, and disassembly. Curr Biol.

[11] Giannone G, Dubin-Thaler BJ, Döbereiner HG, Kieffer N, Bresnick AR, Sheetz MP (2004). Periodic lamellipodial contractions correlate with rearward actin waves. Cell.

[12] Bamburg JR, McGough A, Ono S (1999). Putting a new twist on actin: ADF/cofilins modulate actin dynamics. Trends Cell Biol.

[13] Pantaloni D, Le Clainche C, Carlier MF (2001). Mechanism of actin-based motility. Science.

[14] Mizuno K, Okano I, Ohashi K, Nunoue K, Kuma K, Miyata T, Nakamura T (1994). Identification of a human cDNA encoding a novel protein kinase with two repeats of the LIM/double zinc finger motif. Oncogene.

[15] Huang TY, DerMardirossian C, Bokoch GM (2006). Cofilin phosphatases and regulation of actin dynamics. Curr Opin Cell Biol.

[16] Toshima J, Toshima JY, Amano T, Yang N, Narumiya S, Mizuno K (2001). Cofilin phosphorylation by protein kinase testicular protein kinase 1 and its role in integrin-mediated actin reorganization and focal adhesion formation. Mol Biol Cell.

[17] Edwards DC, Sanders LC, Bokoch GM, Gill GN (1999). Activation of LIM-kinase by Pak1 couples Rac/Cdc42 GTPase signaling to actin cytoskeletal dynamics. Nat Cell Biol.

[18] Agnew BJ, Minamide LS, Bamburg JR (1995). Reactivation of phosphorylated actin depolymerizing factor and identification of the regulatory site. J Biol Chem.

[19] Niwa R, Nagata-Ohashi K, Takeichi M, Mizuno K, Uemura T (2002). Control of actin reorganization by slingshot, a family of phosphatases that dephosphorylate ADF/cofilin. Cell.

[20] Gohla A, Birkenfeld J, Bokoch GM (2005). Chronophin, a novel HAD-type serine protein phosphatase, regulates cofilin-dependent actin dynamics. Nat Cell Biol.

[21] Bernstein BW, Bamburg JR (2010). ADF/cofilin: a functional node in cell biology. Trends Cell Biol.

[22] Mizuno K (2013). Signaling mechanisms and functional roles of cofilin phosphorylation and dephosphorylation Cell Signal..

[23] Wang Y, Shibasaki F, Mizuno K (2005). Calcium signal-induced cofilin dephosphorylation is mediated by slingshot via calcineurin. J Biol Chem.

[24] Zhao JW, Gao ZL, Ji QY, Wang H, Zhang HY, Yang YD, Xing FJ, Meng LJ, Wang Y (2012). Regulation of cofilin activity by CamKII and calcineurin. Am J Med Sci.

[25] Huang S, Robinson RC, Gao LY, Matsumoto T, Brunet A, Blanchoin L, Staiger CJ (2005). Arabidopsis Villin1 generates actin filament cables that are resistant to depolymerization. Plant Cell.

[26] Schmoller KM, Semmrich C, Bausch AR (2011). Slow down of actin depolymerization by cross-linking molecules. J Struct Biol.

[27] Mooren OL, Kotova TI, Moore AJ, Schafer DA (2009). Dynamin2 gtpase and cortactin remodel actin filaments. J Biol Chem.

[28] Vuadens F, Rufer N, Kress A, Kress A, Corthésy P, Schneider P, Tissot JD (2004). Identification of swiprosin 1 in human lymphocytes. Proteomics.

[29] Kroczek C, Lang C, Brachs S, Grohmann M, Dütting S, Schweizer A, Nitschke L, Feller SM, Jäck HM, Mielenz D (2010). Swiprosin-1/EFHD2 controls B cell receptor signaling through the assembly of the B cell receptor, Syk, and phospholipase C gamma2 in membrane rafts. J Immunol.

[30] Dütting S, Brachs S, Mielenz D (2011). Fraternal twins: Swiprosin-1/EFhd2 and Swiprosin-2/EFhd1, two homologous EF-hand containing calcium binding adaptor proteins with distinct functions. Cell Commun Signal.

[31] Vega IE, Traverso EE, Ferrer-Acosta Y, Matos E, Colon M, Gonzalez J, Dickson D, Hutton M, Lewis J, Yen SH (2008). A novel calcium-binding protein is associated with tau proteins in tauopathy. J Neurochem.

[32] Thylur RP, Kim YD, Kwon MS, Oh HM, Kwon HK, Kim SH, Im SH, Chun JS, Park ZY, Jun CD (2009). Swiprosin-1 is expressed in mast cells and up-regulated through the protein kinase C beta I/eta pathway. J Cell Biochem.

[33] Avramidou A, Kroczek C, Lang C, Schuh W, Jäck HM, Mielenz D (2007). The novel adaptor protein Swiprosin-1 enhances BCR signals and contributes to BCR-induced apoptosis. Cell Death Differ.

[34] Ramesh TP, Kim YD, Kwon MS, Jun CD, Kim SW (2009). Swiprosin-1 regulates cytokine expression of human mast cell line HMC-1 through actin remodeling. Immune Netw.

[35] Kim DJ, Kim SH, Lim CS, Choi KY, Park CS, Sung BH, Yeo MG, Chang S, Kim JK, Song WK (2006). Interaction of SPIN90 with the Arp2/3 complex mediate lamellipodia and actin comet tail formation. J Biol Chem.

[36] Blagoev B, Ong SE, Kratchmarova I, Mann M (2004). Temporal analysis of phosphotyrosine-dependent signaling networks by quantitative proteomics. Nat Biotechnol.

[37] Hinz B, Alt W, Johnen C, Herzog V, Kaiser HW (1999). Quantifying lamella dynamics of cultured cells by SACED, a new computer-assisted motion analysis. Exp Cell Res.

[38] Bryce NS, Clark ES, Leysath JL, Currie JD, Webb DJ, Weaver AM (2005). Cortactin promotes cell motility by enhancing lamellipodial persistence. Curr Biol.

[39] Kiuchi T, Ohashi K, Kurita S, Mizuno K (2007). Cofilin promotes stimulus-induced lamellipodium formation by generating an abundant supply of actin monomers. J Cell Biol.

[40] Yamakita Y, Ono S, Matsumura F, Yamashiro S (1996). Phosphorylation of human fascin inhibits its actin binding and bundling activities. J Biol Chem.

[41] Ono S, Yamakita Y, Yamashiro S, Matsudaira PT, Gnarra JR, Obinata T, Matsumura F (1997). Identification of an actin binding region and a protein kinase C phosphorylation site on human fascin. J Biol Chem.

[42] Ghosh M, Song X, Mouneimne G, Sidani M, Lawrence DS, Condeelis JS (2004). Cofilin promotes actin polymerization and defines the direction of cell motility. Science.

[43] Nishita M, Tomizawa C, Yamamoto M, Horia Y, Ohashi K, Mizuno K (2005). Spatial and temporal regulation of cofilin activity by LIM kinase and Slingshot is critical for directional cell migration. J Cell Biol.

[44] Delorme V, Machacek M, DerMardirossian C, Anderson KL, Wittmann T, Hanein D, Waterman-Storer C, Danuser G, Bokoch GM (2007). Cofilin activity downstream of Pak1 regulates cell protrusion efficiency by organizing lamellipodium and lamella actin networks. Dev Cell.

[45] Chan AY, Bailly M, Zebda N, Segall JE, Condeelis JS (2000). Role of cofilin in epidermal growth factor-stimulated actin polymerization and lamellipod protrusion. J Cell Biol.

[46] Zhang XF, Hyland C, Van Goor D, Forscher P (2012). Calcineurin-dependent cofilin activation and increased retrograde actin flow drive 5-HT-dependent neurite outgrowth in Aplysia bag cell neurons. Mol Biol Cell.

[47] Takemura M, Mishima T, Wang Y, Kasahara J, Fukunaga K, Ohashi K, Mizuno K (2009). Ca2+/calmodulin-dependent protein kinase IV-mediated LIM kinase activation is critical for calcium signal-induced neurite outgrowth. J Biol Chem.

