# Supplementary material for: Swiprosin-1 modulates actin dynamics by regulating the F-actin accessibility to cofilin
Source: Cell Mol Life Sci. 2013 Aug 21;70(24):4841–54. doi: 10.1007/s00018-013-1447-5 (PMC3830201; doi:10.1007/s00018-013-1447-5)

**SUPPLEMENTARY MATERIAL**

Swiprosin-1 Modulates Actin Dynamics by Regulating the F-actin Accessibility to Cofilin

^1^Yun Hyun Huh, ^1,2^So Hee Kim, ^1^Kyoung-Hwun Chung, ^1,2^Sena Oh, ^1,2^Min-Sung Kwon, ^2^Hyun-Woo Choi, ^3^Sangmyung Rhee, ^4^Je-Hwang Ryu, ^2^Zee Yong Park , ^1,2^Chang-Duk Jun

and ^1,2^Woo Keun Song¶

^1^Bio Imaging and Cell Dynamics Research Center, ^2^School of Life Sciences,

Gwangju Institute of Science and Technology, Gwangju, 500-712, Korea

^3^ School of Biological Sciences, Joong Ang University, Seoul, 156-756, Korea

^4^ Research Center for Biomineralization Disorders and Dental Science Research Institute, School of Dentistry, Chonnam National University, Gwangju, 500-757, Korea

Corresponding author :

Woo Keun Song, Ph.D.

E-mail: wksong@gist.ac.kr

**MATERIALS AND METHODS**

*Reverse transcription-polymerase chain reaction (RT-PCR) and quantitative real-time polymerase chain reaction (qRT-PCR)*

Total RNA was isolated using TRI reagent (Molecular Research Center, Cincinnati, OH, USA) and reverse-transcribed using TOPscript RT drymix (Enzynomics, Daejeon, Korea). The resulting cDNA was subjected to PCR using Taq polymerase (iNtRON BioTechnology, Seongnam, Korea) with the following primers: 5’-cggcagggatggcttcat-3’ (sense) and 5’-ttggcacccttaacgccc-3’ (antisense) for *Swiprosin-1*; 5’-tcttcaatccctacaccg-3’ (sense) and 5’-tggaaaatgagcaggaac-3’ (antisense) for *Swiprosin-2*; 5’-tcaccatcttccaggagcga-3’ (sense) and 5’-cacaatgccgaagtggtcgt-3’ (antisense) for *glyceraldehyde-3-phosphate dehydrogenase (GAPDH)*. qRT-PCR was performed using an iCycler^TM^ thermal cycler (Bio-Rad, Hercules, CA, USA) and SYBR Premix ExTaq (TaKaRa Bio Inc, Shiga, Japan). All qRT-PCR reactions were duplicated for each independent experiment, and the amplification signal from the target gene was normalized to the *GAPDH* signal in the same reaction.

***SUPPLEMENTARY FIGURE LEGENDS***

**Supplementary Figure S1.** Specificity of the rabbit anti-Swiprosin-1 antibody. (a) B16F10 cells were transfected with psi-RNA-*GFP*-control or psi-RNA-*GFP-Swiprosin-1* for 24 h. Knockdown of Swiprosin-1 was determined by immunoblotting with the anti-Sw1-R antibody, indicating that the newly generated antibody is reactive for immunoblotting in mouse B16F10 cells. (b) Lysates of B16F10 cells were immunoprecipitated with an anti-Sw1-R antibody, and immunoblotted with anti-Sw1-G and mouse anti-Sw2 antibodies.

**Supplementary Figure 2.** Knock-down of Swirpsoin-1. B16F10 cells were transfected with psi-RNA-*GFP*-control or various clones (sh1-1, sh1-2, sh2-3, and sh2-4) of psi-RNA-*GFP-Swiprosin-1* for 24 h. Knockdown effects of *Swiprosin-1* shRNAs were analyzed using RT-PCR and Western blotting (left panel) and quantitative real-time PCR (qPCR) (right panel).

**Supplementary Figure 3.** Swiprosin-1 is involved in membrane dynamics. B16F10 cells were transfected with empty *GFP* vector or *GFP-Swiprosin-1* for 24 h. Kymographic images were created from 60 frames of time-lapse images (every 5 s for 5 min) at each region indicated by the 20-μm *yellow bars* drawn in the direction of the cell protrusions. *Scale bar* in the cell images is 10 μm; *scale bars* in kymographic images are 5 μm and 1 min, respectively.

**Supplementary Figure 4.** Swiprosin-1 is not involved in actin polymerization. The reaction mixture for actin polymerization assay contained 5 μM pyrene-actin, 50 nM Arp2/3 complex, 50 nM WASP-VCA and 1 μM GST or GST-Swprosin-1. To examine whether Swiprosin-1 plays a role as a cofactor on actin polymerization it was assessed by pyrene-actin assays. The kinetics was monitored by measuring the fluorescence intensity of pyrene-F-actin.

**Supplementary Figure 5.** HEK293T cells were transfected with myc-tagged *Swiprosin-1* or empty vector (Ev), after which the lysates were immunoprecipitated (IP) with anti-myc antibody and immunoblotted with anti-myc, -actin, -cofilin or -phospho-cofilin antibody.

**Supplementary Figure 6.** Disintegration of F-actin by Cofilin. G-actin (5 μM) and GST or GST‑Swiprosin-1 (2 μM) were incubated with the indicated concentrations of cofilin. The reactants were then ultracentrifuged, and the supernatant (S) and pellet (P) fractions were separated by SDS-PAGE.


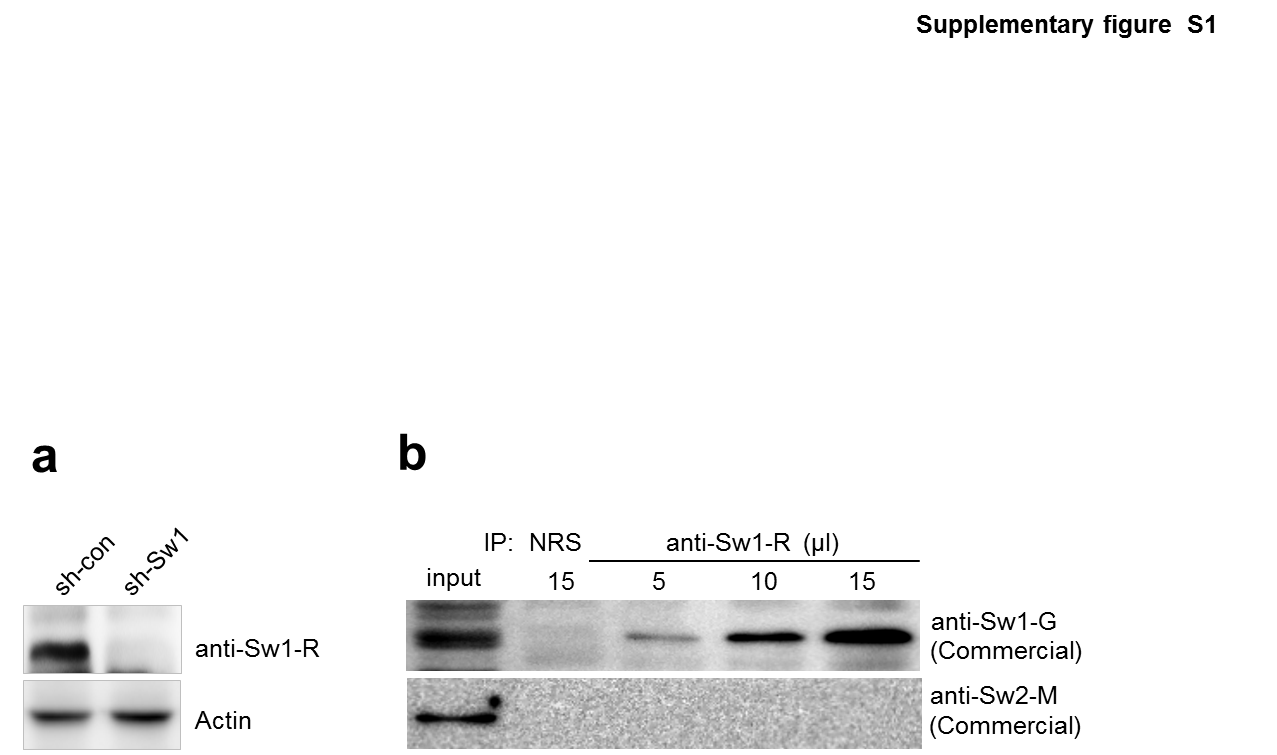


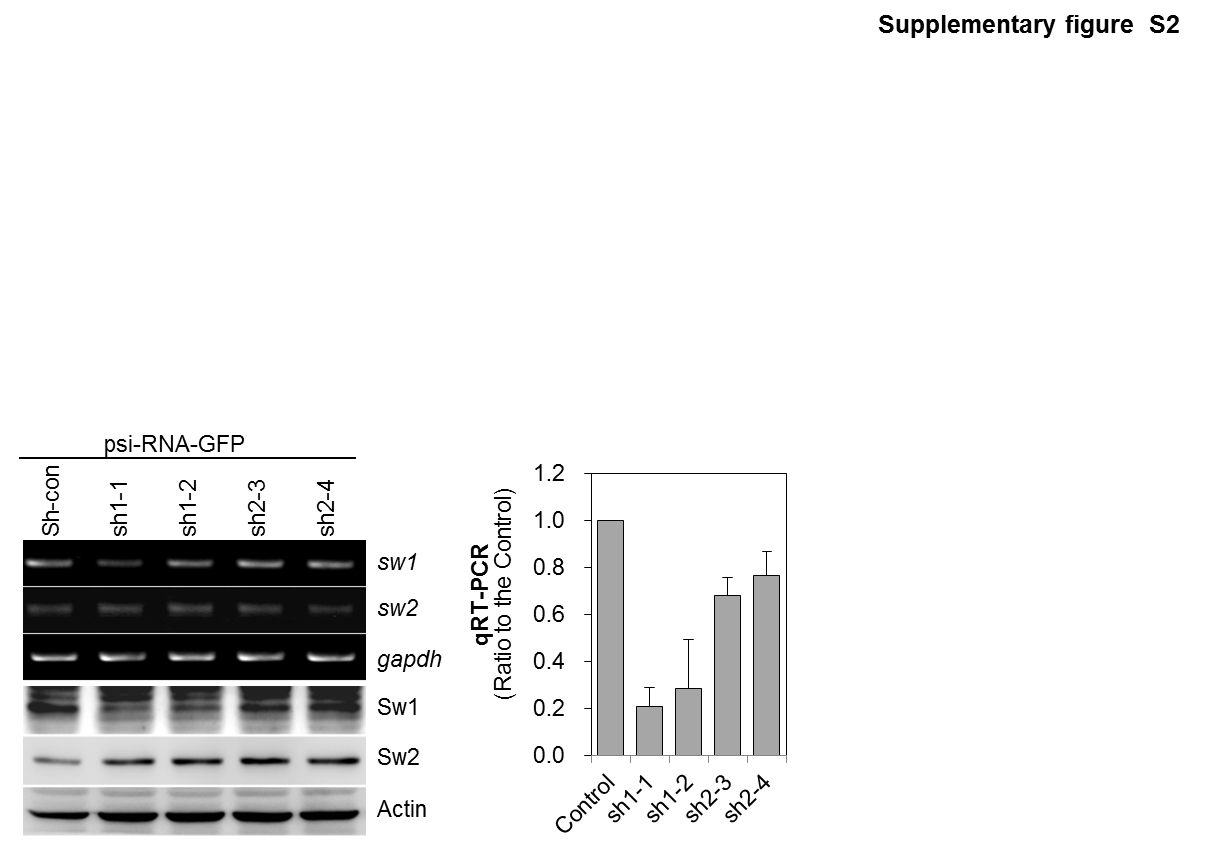


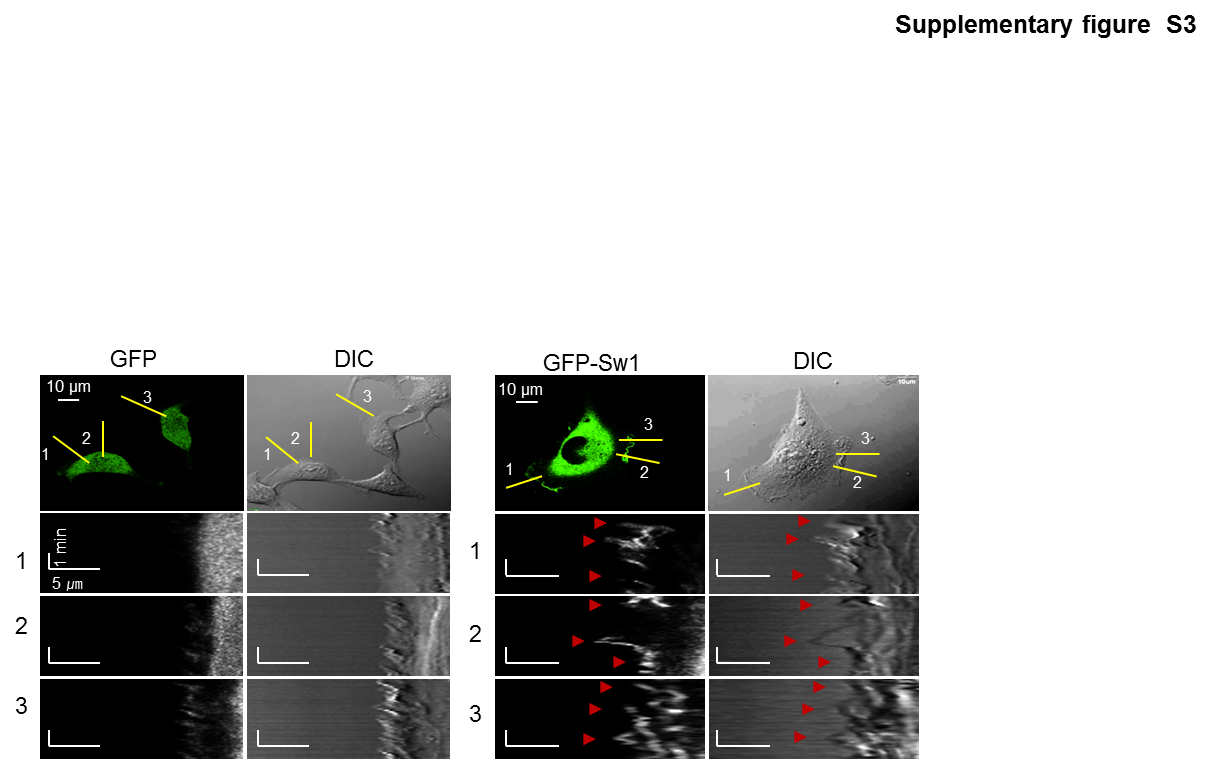


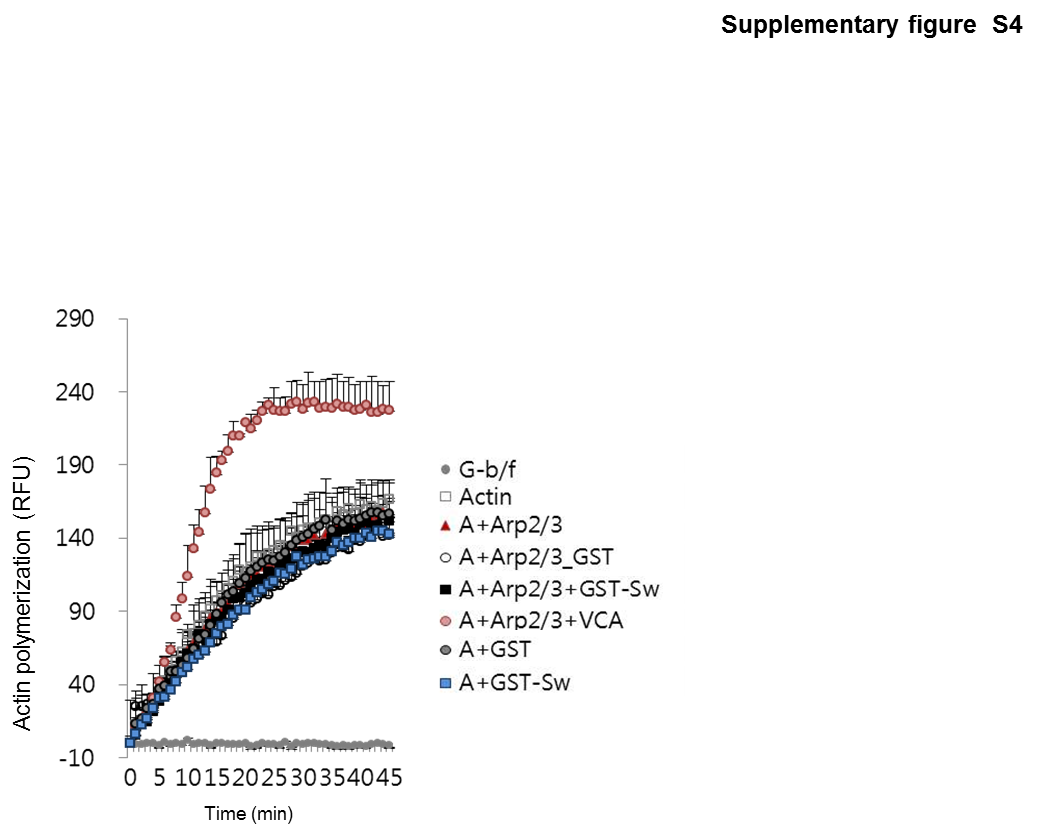


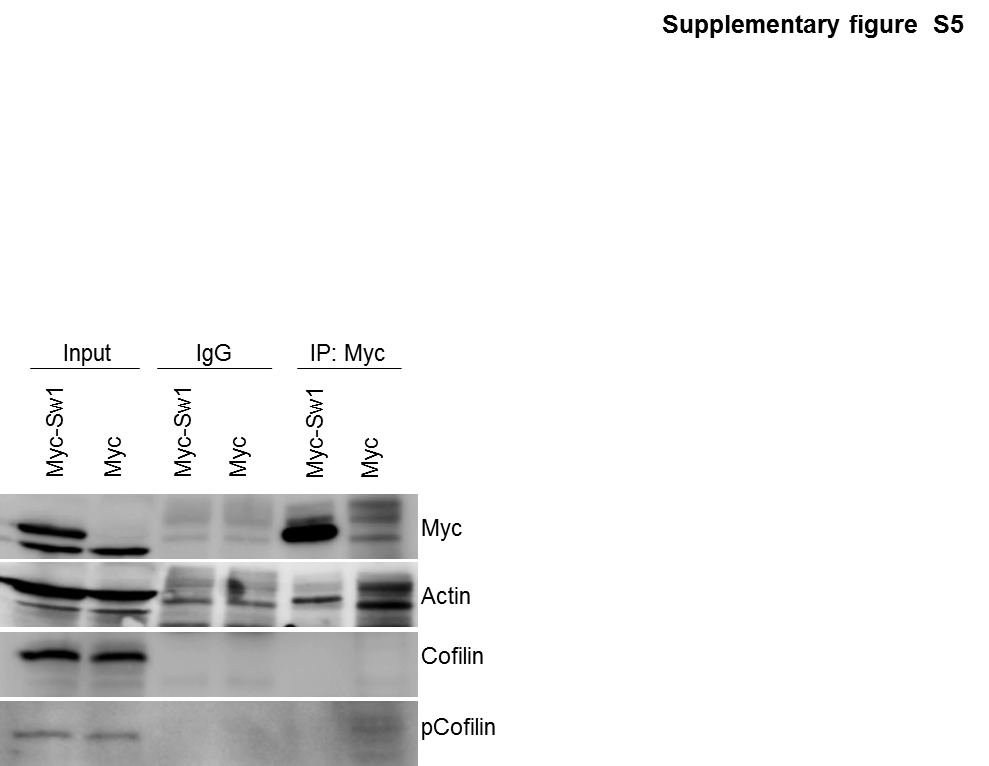


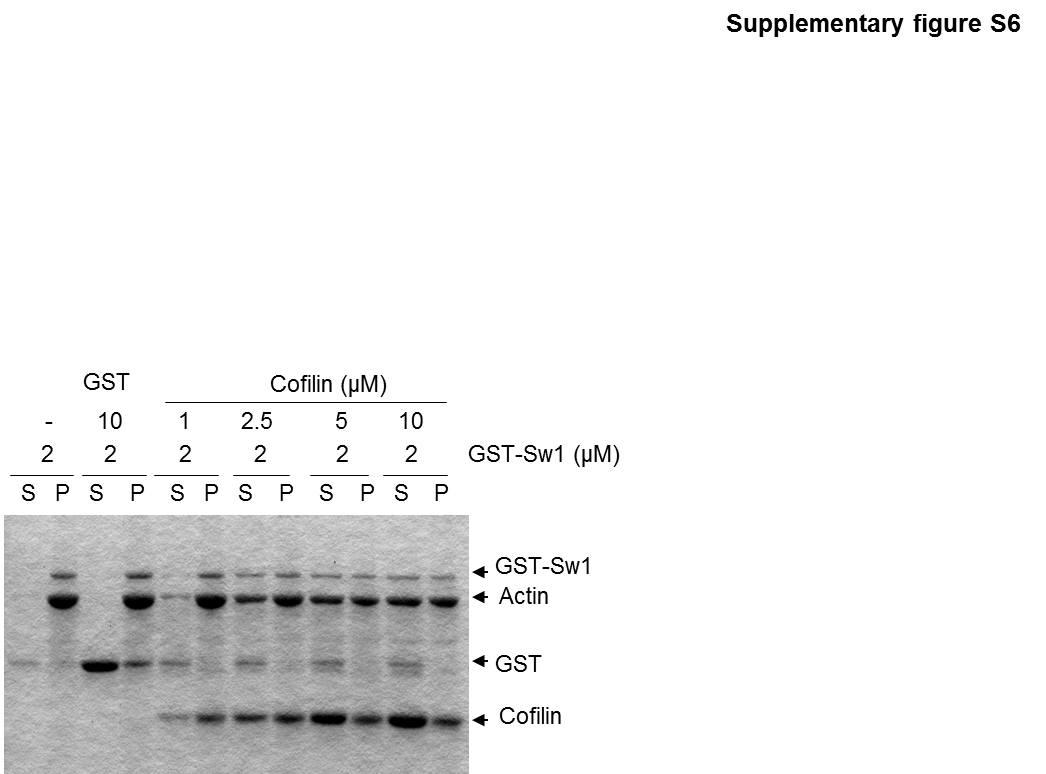

Supplement: Supplementary file 1 — Supplementary material (DOCX 806 kb) [file 18_2013_1447_MOESM1_ESM.docx]
